# Collagen-based injectable and self-healing hydrogel with multifunction for regenerative repairment of infected wounds

**DOI:** 10.1093/rb/rbad018

**Published:** 2023-03-07

**Authors:** Haojie Gu, Han Li, Liren Wei, Jian Lu, Qingrong Wei

**Affiliations:** National Engineering Research Center for Biomaterials (NERCB), Sichuan University, Chengdu 610065, P.R. China; College of Biomedical Engineering, Sichuan University, Chengdu 610065, P.R. China; National Engineering Research Center for Biomaterials (NERCB), Sichuan University, Chengdu 610065, P.R. China; College of Biomedical Engineering, Sichuan University, Chengdu 610065, P.R. China; School of Life Science and Engineering, Southwest University of Science and Technology, Mianyang 621000, P.R. China; National Engineering Research Center for Biomaterials (NERCB), Sichuan University, Chengdu 610065, P.R. China; College of Biomedical Engineering, Sichuan University, Chengdu 610065, P.R. China; National Engineering Research Center for Biomaterials (NERCB), Sichuan University, Chengdu 610065, P.R. China; College of Biomedical Engineering, Sichuan University, Chengdu 610065, P.R. China

**Keywords:** collagen, injectable and self-healing, hydrogel, regenerative repair, wounds

## Abstract

At present, the development trend of dressing materials is being multifunctional for convenient and long-term nursing care process of some complicated wounds. Here, basing on the theory of wound moist healing, an injectable and self-healing hydrogel comprising of collagen (COL), chitosan (CS) and oxidation modified Konjac glucomannan (OKGM), which acts as a macromolecular cross-linker to construct dynamic Schiff-base bonds was smartly designed. The strategy of introducing the silver nanoparticles (Ag NPs) into the COL–CS–OKGM hydrogel matrix achieved a markedly enhanced antibacterial activity derived from the synergistical effect between the Ag^+^ and the mild photothermal efficacy of Ag NPs, which also improved the local capillary blood circulation of the wound area to further facilitate wound healing process. The excellent syringeability and self-healing behaviors endowed the COL–CS–OKGM–Ag hydrogel with self-adapting ability for the wounds with irregular and large area needing frequent applying and changing without secondary injuries. *In vitro* and *in vivo* evaluations verified that so-designed COL–CS–OKGM–Ag hydrogel also with hemostatic performance is a promising multifunctional dressing for the treatments of infected wound with not only good biocompatibility and convenient use, but also with desired regenerative healing prognoses benefited from hydrogel moist environment and physiotherapy.

## Graphical Abstract

**Figure:**
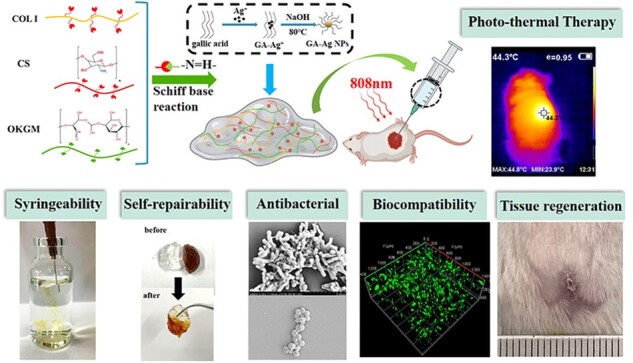

## Introduction

Healthy skin is not only a physical barrier but also an important immune organ, protecting internal organs from pathogen invasion and other external threats [1]. Wound infections are usually caused by severe microbial invasion derived from skin tissue damage, which can produce some inflammation that significantly increases the infected wound related diseases and reduces the quality of wound healing [2]. Additionally, various complications from wound infection with impaired healing could be life-threatening in some cases [3]. Traditional therapeutic materials such as gauze easily dehydrates and scabs the wound, increasing pain and infection rate, and also cannot conducive to the migration of epithelial cells, consequently resulting in poor-quality healing [4]. To make matters worse, the strong attachment of gauze to wound usually causes unpleasant pain and further damage when replacing the dressing [5].

In recent decades, studies have found that a moderately moist microenvironment is more suitable and ideal for the regenerative repair of the wound tissues, minimizing or even avoiding scarring repair [6].

Owning to possessing the excellent properties such as biomimetic porosity and good water retention for creating a local moist ambience etc., hydrogels are desirable materials for facilitating the regenerative healing of wound tissues [7]. Additionally, hydrogels are also widely utilized as efficient carriers for functional nanoparticles and drug delivering, particularly as new platform materials for surgical dressing in biomedical applications [8, 9].

Although hydrogels are used as a carrier of antibacterial drugs for wound infection treatments, the abuse of antibiotics and the drug resistance of bacteria have greatly weakened the treatment effect of infected wounds in these year [10]. Near-infrared (NIR) laser-triggered photothermal therapy (PTT) based on nanoparticle has become one of the most effective antibacterial strategies for its few side effects, low systemic toxicity, high spatial resolution and tissue penetration depth [11]. In addition, NIR laser can focus on wound area to improve local blood circulation and alleviate tissue inflammation [12]. More importantly, the antimicrobial efficacy of PTT is not limited by bacterial resistance when compared with conventional antibiotic treatment. Used alone, however, PTT easily causes a certain degree of damage to healthy skin tissue around the wound because the temperature required for killing bacteria is relatively high [13]. An option to overcome this defect is to develop a synergy therapy based on PTT, which integrates the advantages of single therapy, to strengthen the antibacterial effect and minimize possible damage in normal cells. This synergy not only can generate more efficient antibacterial behavior but also can control area temperature at 40–45°C to make a favorable environment for wound healing. Such mild photothermal physiotherapy also facilitates promoting local blood circulation and angiogenesis of the wound tissue [14].

Hydrogels are appropriate to be used for synergistic therapy including wound healing [15]. Especially injectable self-healing hydrogels have become concerns because of its convenient operation, flexible application and self-repairing after damage by external forces. Such kind of hydrogel as dressing is based on the significant theory of moist wound healing [16], and therefore quite suitable for clinical uses aiming at various wounds including infected wounds [17] due to maintaining a necessary condition of sustained moist environment for scheme regenerative wound healing [18]. Both collagen (COL) and chitosan (CS) are natural biomacromolecules developed as the ideal biomedical materials with excellent biocompatibility. As a main component of mammalian tissue extracellular matrix (ECM), collagen has good adhesion to various cells and promotion of rapid angiogenesis in skin tissue and corneal [19, 20]. Chitosan is characteristic of the properties of antibacterial activity and non-toxicity [21]. Collagen and chitosan have a good synergistic compatibility, and a complex of these two macromolecules is expected to mimic the composition of ECM [22], which has been demonstrated to have new or improved properties [23].

However, most of the traditional collagen–chitosan composite hydrogels relying on collagen macromolecular self-assembly to achieve gelation, which are normally permanent network produced by irreversible interactions between molecular chains [24]. For these COL–CS hydrogels, there are some shortcomings such as too short operation window period, an inconvenience of formulating dressing material upon applying and the difficulty to maintain complete cover on wound area due to their absence of self-healing properties after being cracked [25]. Konjac glucomannan (KGM) is another natural polysaccharide as the main component of the tubers and roots of Amorphophallus konjac plants, which is recognized as safe material by Food and Drug Administration (FDA) regulations for its healthcare benefits [26]. Oxidation modified konjac glucomannan (OKGM) is usually applied as a macromolecular cross-linker agent for positive polyelectrolyte such as chitosan to construct injectable and self-healing hydrogels for minimally invasive treatment of cancer [27] and irregular or chronic wounds [28].

Hence, basing on our previous work [27], and inspired by the photothermal effect along with the inherent antibacterial properties of silver nanoparticles, we synthesized gallic acid-modified silver nanoparticles (GA–Ag NPs) by one-step method firstly, then we dispersed these silver nanoparticles into OKGM crosslinked collagen–chitosan composite hydrogel (COL–CS–OKGM–Ag), in which the principle of Schiff base reactions was utilized to design a typical injectable and self-healing hydrogel as wound dressing with hemostasis and antibacterial activities. When such hydrogel was irradiated under NIR light, the local temperature of wound area can be elevated to cause the destruction of bacterial cell membranes and protein denaturation [29]. Simultaneously, the mild temperature also can facilitate the regeneration of capillaries in wound area. Additionally, the stimulation of NIR laser irradiation could further stimulate the release of Ag^+^, thus increasing the interaction with the protein sulfhydryl groups on the damaged membrane and inducing the death of bacteria [30]. Consequently, so-designed hydrogel on one hand can perform a dual antibacterial strategy for the treatment of infected wounds. On the other hand, it can provide an indispensable moist environment for wound tissue healing by regeneration other than cicatrix formation. Significantly, the injectable and self-healing behaviors of COL–CS–OKGM–Ag composite hydrogel significantly expand the function and scene scope in clinical applications.

## Materials and methods

### Materials

Medical-grade collagen (Type I) with high purity was extracted from calf skin by modified method in our laboratory basing on Miller description [31]. Chitosan (CS, molecular weight (Mw): 310–375 kDa, degree of deacetylation >75%) was obtained from Sigma-Aldrich. KGM powder (purity ≥ 90%, 200-mesh) was purchased from Sheli Ltd (Hongkong, China) and further purified upon receiving. Sodium periodate (NaIO_4_, ≥99.5%) were provided by Aladdin (Shanghai, China). Chloral hydrate and fluorescein diacetate (FDA) were purchased from Sigma-Aldrich (St Louis, MO, USA). Phosphate buffered saline (PBS) and all sterile consumables used in cell experiments were obtained from Corning (USA). CCK-8 kits were provided by Beyotime (Shanghai, China). Bovine serum albumin 4% and paraformaldehyde solution were supplied by Solarbio (Beijing, China). The ultrapure water from a Milli-Q system (Millipore, Billerica, MA, USA) was used in all procedures of our experiments.

### Preparation of GA–Ag NPs and OKGM

Ten milliliters of saturated gallic acid solution were added to 80 ml of silver nitrate aqueous solution (0.01 M) with stirring vigorously. Then the temperature of the mixture was raised to 80°C, while aqueous NaOH solution (1 M) was added dropwise to adjust the pH to about 11 for the solution, followed by a stirring of 30 min in dark. The obtained mixture was dialyzed (molecule cutoff 8000–12 000) for 48 h after cooling to room temperature and stored. To embed these silver nanoparticles into the hydrogel prepared later, the silver nanoparticle suspension was concentrated by swirling evaporation to obtain the final suspension with a concentration of about 1290 µg/ml Ag NPs, which was used for following experiments.

A certain amount of KGM aqueous solution (0.8%, w/v) was mixed with sodium periodate aqueous solution, whose final concentration in the mixture was 18 mM. Then the mixture was stirred at room temperature in dark for 12 h, whereafter 200 µl of ethylene glycol was added to terminate the reaction. The obtained product was dialyzed (molecule cut off 8000–12 000) for 72 h with following concentration by reduced-pressure distillation to obtain the needed OKGM solution.

### Preparation of COL–CS–OKGM–Ag hydrogel

Five milliliters of collagen acid solution (1%, w/v) and 5 ml of CS acid solution (2%, w/v) were mixed at low temperature maintained by ice bath with stirring at 500 rpm for 10 min. A certain amount of OKGM solution (2.4%, w/v) was added dropwise under stirring. Next, a certain amount of concentrated silver nanoparticle suspension was then added to make the final concentration of silver nanoparticles in the hydrogel to be 0, 50, 100, 150 and 200 µg/ml, which are labeled as COL–CS–OKGM hydrogel or COL–CS–OKGM–Ag hydrogels for all the following references. Subsequently, the pH value of the solution was adjusted to about 6–7 with 1 M NaOH to obtain a composite hydrogel of COL–CS–OKGM–Ag, which was poured into a clean centrifuge tube and stored at 4°C.

Characterizations of the COL–CS–OKGM–Ag hydrogel were detailed in Supplementary data.

### *In vivo* antibacterial and wound healing

All *in vivo* experiments were approved by the Institutional Animal Care and Use Committee (IACUC) of Sichuan University and carried out in accordance with the guidelines for animal rights formulated by Sichuan University, which comply with EU Directive 2010/63/EU for animal experiments. All animals were housed under standard specific pathogen-free animal facilities. The relevant details were available in the Supplementary data.

### Statistical analysis

All data were presented as mean ± SD of at least three representative trials, with the use of one-way statistical analysis of variance. The significance levels were set at *P *<* *0.05 (*), *P *<* *0.01 (**), *P *<* *0.001 (***); and n.s. represents no significant difference.

## Results and discussion

### Synthesis and characterization of the GA–Ag nanoparticles

**Figure 1.**
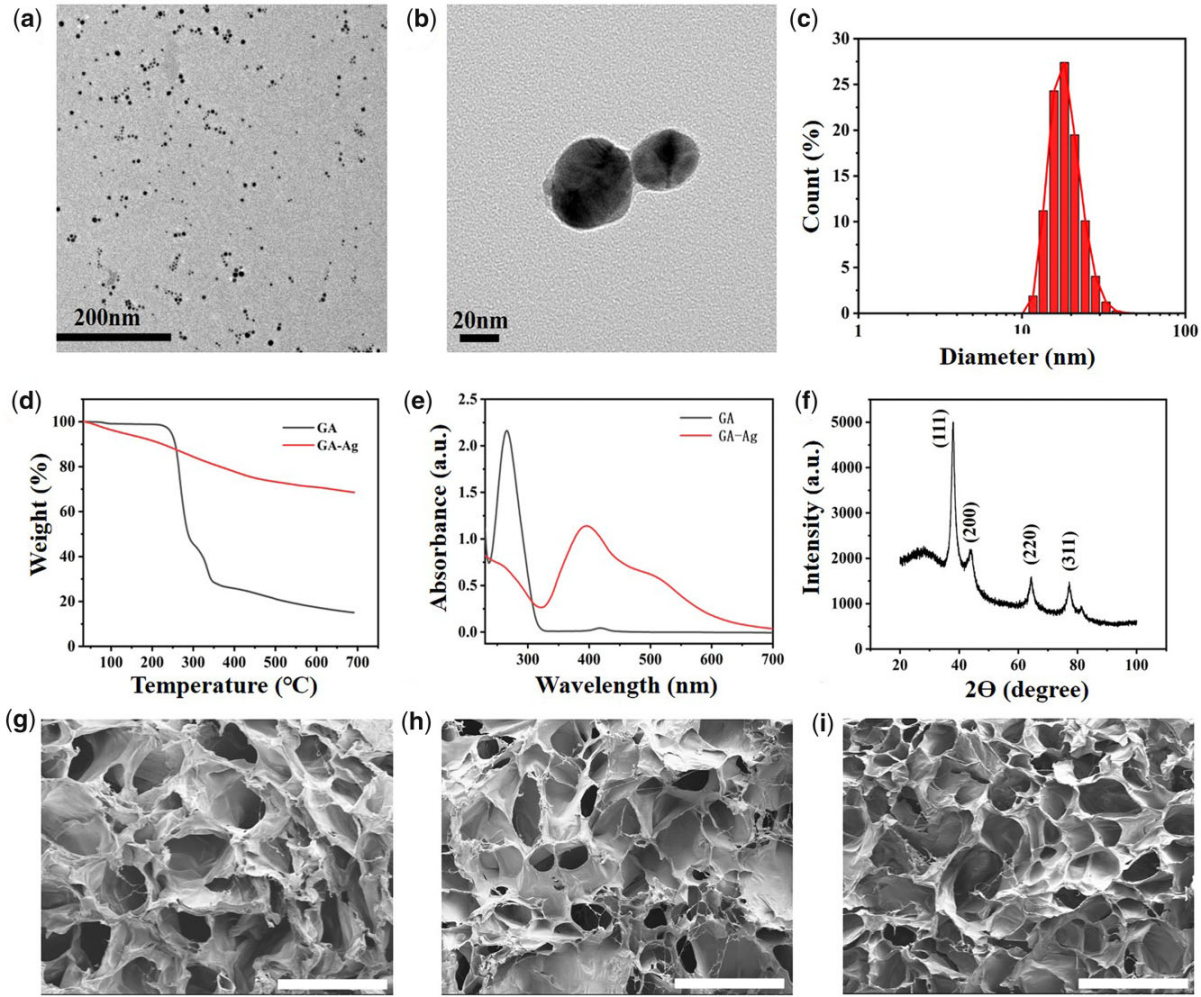
TEM images of GA–Ag NPs under low power (**a**) and at high magnification (**b**). Size distribution of GA–Ag NPs (**c**). TGA (**d**), ultraviolet spectrophotometer (**e**) and XRD profile (**f**) of GA–Ag NPs. SEM images of CS-OKGM hydrogel (**g**), COL–CS–OKGM hydrogel (**h**) and COL–CS–OKGM–Ag hydrogel (**i**). Bar = 500 μm.

In view of the advantages of good antibacterial potency, antioxidant property and biocompatibility [32], gallic acid was chosen as a reducing agent for silver nanoparticle synthesis from silver ions. GA–Ag NPs are spherical in shape, with good stability and polydispersity (Fig. 1a) because of the adsorption of gallic acid molecules on the surface of the silver nanoparticles (Fig. 1b). Dynamic light scattering analyses revealed an average diameter of 18 nm with narrow size distribution of GA–Ag nanoparticles in water (Fig. 1c).

The proportion GA in GA–Ag nanoparticles was estimated to be ∼37% from the thermogravimetric analysis (TGA) curve (Fig. 1d). The GA–Ag NPs have two peaks under UV spectrophotometer (Fig. 1e). The one peak at 396 nm is the absorption peak of silver nanoparticles [33], the other peak at 262 nm indicates the existence of GA molecules on the surface of the silver nanoparticles, which agrees with the absorption peak of pure GA [34]. X-ray diffraction (XRD) analysis showed that the crystal structure of GA–Ag NPs was face-centered cubic (Fig. 1f). There were four typical diffraction peaks at 2θ of 37.98° (111), 44.02° (200), 64.22° (220) and 77.22° (311), which completely conform to the Joint Committee on Powder Diffraction Standards (JCPDS, file no. 01-1164).

**Figure 2.**
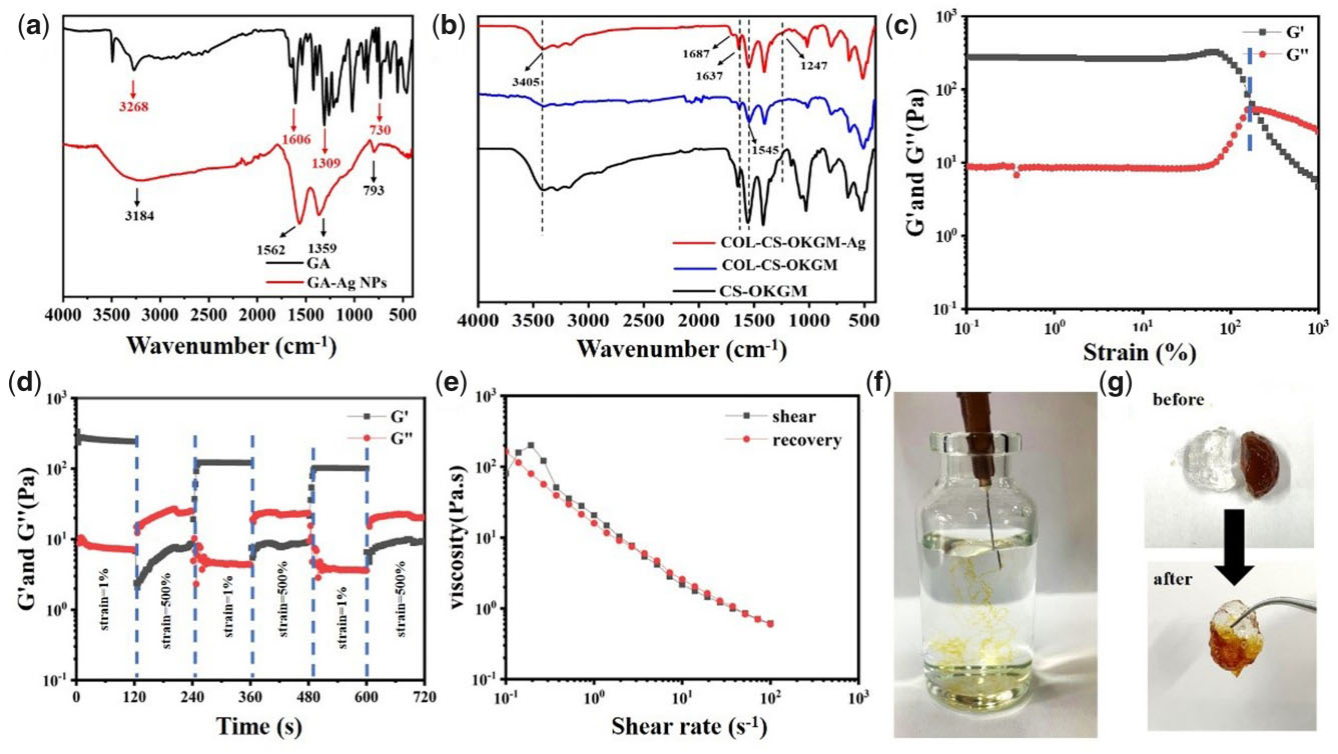
FTIR spectra of (**a**) GA–Ag NPs and (**b**) hydrogels. (**c**) Strain amplitude sweep and (**d**) step–strain measurements of COL–CS–OKGM–Ag hydrogel. Viscosity and shear-thinning behavior of COL–CS–OKGM–Ag hydrogel (**e**). Photographs of hydrogel injection via 26G syringe needle (**f**) and (**g**) fusion of two separated hydrogels showing the easy self-healing behavior of COL–CS–OKGM–Ag hydrogel.

The FTIR spectra of gallic acid modified silver nanoparticles and pure gallic acid are shown in Fig. 2a. The peak at 793 cm^−1^ is ascribed to the benzene ring vibration in gallic acid molecules on GA–Ag NPs. The peak at 1359 and 1562 cm^−1^ correspond to the C=C stretching vibrations in the benzene rings. The functional groups of oxhydryl deriving the peak at 3184 cm^−1^ belong to the carboxyl or phenolic hydroxyl groups. These evidences indicate a successful modification for Ag NPs with GA [34, 35].

### Synthesis of injectable and self-healing hydrogels basing on collagen and OKGM

In this study, a hydrogel dressing possessing injectable and self-healing properties for surgical applications was designed, integrating various advantages of excellent antibacterial properties, biocompatibility and blood coagulation capacity. Traditionally, the composite of collagen and chitosan has many merits for biomedical applications but forming irreversible hydrogel network and failure to self-repair after destruction limit their further development. In this work, OKGM was selected as a macromolecular cross-linking agent to endow the collagen–chitosan-based hydrogels with injectable and self-healing properties. Aldehyde groups from OKGM and amino groups from collagen and chitosan formed dynamic chemical bonds of Schiff base, which results in dynamic and reversible rather than permanent hydrogel network. Moreover, with temperature increasing to physiological level, collagen macromolecules were activated to occur local self-assembly process. Therefore, a novel type of hydrogel with physicochemical cross-linked double network comprising of dynamic chemical bonds and self-assembly fibrils was constructed.

As shown in Fig. 1g and h, after the incorporation of collagen, the pore morphology of CS-OKGM hydrogel almost remained unchanged, but the pore size was significantly reduced, which contained filamentous fibrils mainly generated by self-assembly behavior of part collagen. For the COL–CS–OKGM–Ag composite hydrogel containing 200 μg/ml GA–Ag NPs, its micropores seems to become further small and uniform (Fig. 1i), whose swelling rate of lyophilized sponge shape reached about 1675% at 2 min in PBS (Supplementary Fig. S1), which could facilitate absorbing a large amount of wound effusion. The anti-dehydration performance of the hydrogels was evaluated in Supplementary Fig. S2, demonstrating these hydrogels could provide a relatively humid environment for up to 9 h, which was conductive to wound healing. Further, the anti-dehydration time could be further extended by absorbing blood or exudates seeping out of wounds early in healing. The surface elements of COL–CS–OKGM–Ag composite hydrogel were analyzed under energy dispersive spectrometer (Supplementary Fig. S3), whose result indicated that GA–Ag NPs were evenly distributed in the hydrogel matrix.

The chemical structures and characteristic groups of OKGM (Supplementary Fig. S4) and as-prepared hydrogels (Fig. 2b) were verified via FT-IR. Compared with the FTIR spectrum of KGM, the spectrum of OKGM showed two characteristic bands at 1730 and 893 cm^−1^. The one at 1730 cm^−1^ represents the symmetrical vibration band of the aldehyde group [36], while the other one at 830 cm^−1^ is attributed to the hemiacetal structure formed between the aldehyde group and the adjacent hydroxyl group [37]. Meanwhile, the peak at 806 cm^−1^ corresponds to the respiratory vibration peak of the pyran ring in the KGM macromolecular chain, which almost disappeared in the OKGM spectrum, further suggesting the ring-opening oxidation of KGM macromolecules [38]. The Mw of OKGM was determined to be ∼72.8 kDa by gel permeation chromatography (Supplementary Table S1), indicating that OKGM is a typical biological macromolecular crosslinking agent. As shown in Fig. 2b, the peak at 1637 cm^−1^ corresponds to the stretching vibration of C=N [39], which confirms successful construction of the dynamic hydrogel network basing on Schiff-base bonds. The broad band at 3405 cm^−1^ should be attributed to the stretching vibrations of O–H and N–H in chitosan [40]. In the spectra of the COL–CS–OKGM hydrogel, the characteristic peaks at 1687, 1545 and 1247 cm^−1^ correspond to the amide I (C=O stretching), amide II (N–H bending) and amide III (C–N stretching) bands in collagen macromolecules, respectively [41, 42].

### Injectable and self-healing properties of the hydrogel

Hydrogels possessing permanent network formed by irreversible crosslinking could not restore to their previous whole upon a split by external force which exceed their own endurance. As functional wound dressing, the repairable self-healing hydrogels based on Schiff bases have received intensive attentions benefit from the excellent performances of use convenience and versatile clinical services [43, 44]. Rheological recovery test was applied for COL–CS–OKGM–Ag hydrogel to explore its self-healing behavior.

First, strain amplitude sweep tests were performed to confirm the transition point between the liquid phase and solid phase of the hydrogel. As shown in Fig. 2c, at a frequency of 1 Hz, a plateau area was appeared first in the range strain of 1–70%, in where the storage modulus (*G*′) and loss modulus (*G*″) had no obvious changes. As the strain continued to increase, the *G*′ values decreased rapidly and the *G*″ values rose sharply. After the strain went beyond the yield point (197%), the *G*′ values fell to be less than *G*″, indicating that the hydrogel network was broken.

Then the self-healing capability of the hydrogel was evaluated by step strain tests (1 Hz) (Fig. 2d). The *G*′ value was higher than *G*″ in low strain (1%), suggesting the sample was in a hydrogel state at this time. When a high strain (500%) was applied, *G*′ quickly decreased to ∼7 Pa. In this case, the sample underwent a sol–gel transition and presented a viscous liquid state with a *G*′ value lower than *G*″. After experiencing multiple damage-healing cycles later, the storage modulus and loss modulus of the hydrogel were basically unaffected, which is the result of the dynamic formation of Schiff base between the amino groups and the aldehyde groups. These reversible and dynamic bonds derived the mechanical properties of self-healing and syringeability. In addition, the viscosity changes of the hydrogel were monitored by a step-shear measurement at high magnitude shear rate of 100 s^−1^ (Fig. 2e). The viscosity of the hydrogels decreased with the increase of the shear rate, showing a recovery cycle with the shear rate decreasing, which demonstrated a typical shear thinning behavior to simulate the hydrogel state under the injection conditions in needle. By some further intuitive methods, the hydrogel was injected into water through a syringe (Fig. 2f) to observe its injecting behavior in liquid environment and its self-healing capability after incision (Fig. 2g).

### Photothermal effect of COL–CS–OKGM–Ag hydrogels

**Figure 3.**
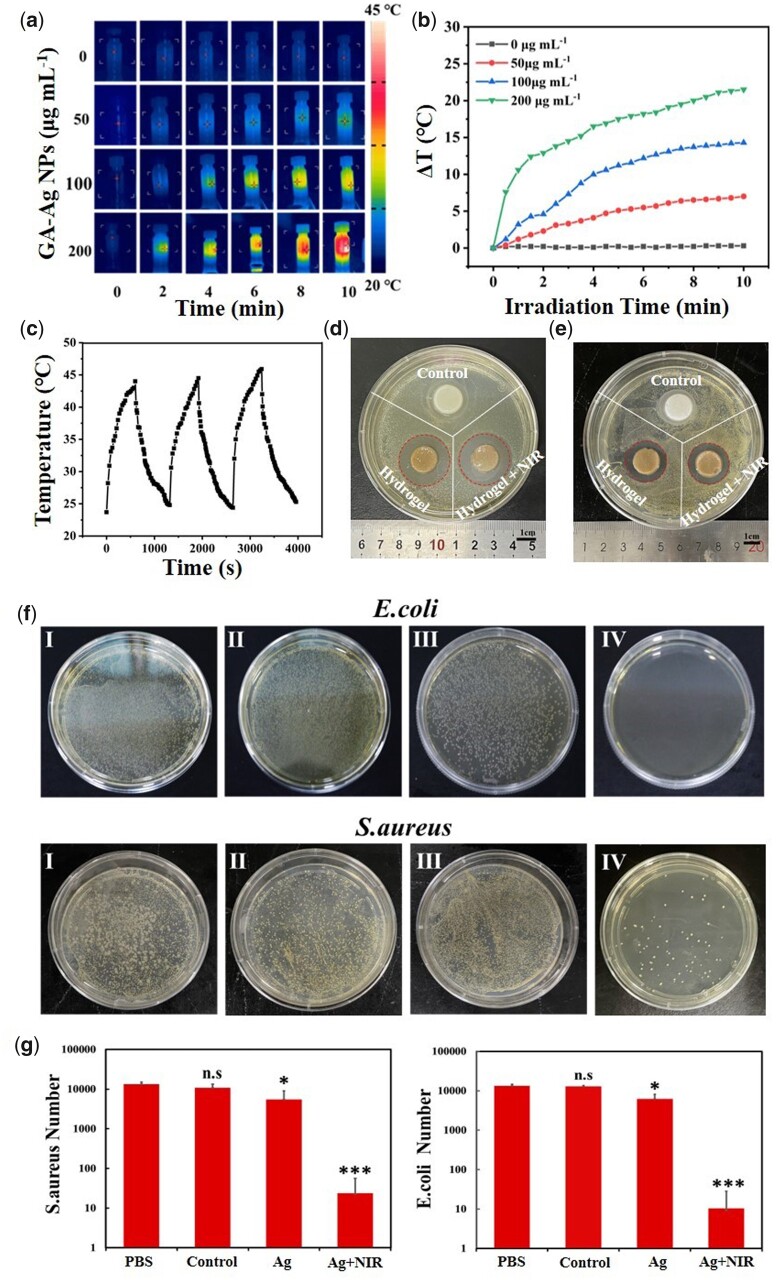
(**a**) Thermal infrared images of hydrogels with different Ag NPs content under 808 nm NIR irradiation. (**b**) Temperature profiles of COL–CS–OKGM–Ag hydrogels with different silver concentrations during the extension of irradiation time. (**c**) Temperature variation of the COL–CS–OKGM–Ag hydrogel containing 200 μg/ml Ag NPs during three on/off laser irradiation cycles (808 nm 2 W/cm^2^). Photograph of differently treated hydrogels incubated at 37°C for 12 h against inhibitory bands of (**d**) *E. coli* and (**e**) *S. aureus*. (**f**) Photographs of *E. coli* and *S. aureus* treated by (I) PBS, (II) COL–CS–OKGM hydrogel, (III) COL–CS–OKGM–Ag hydrogel containing 200 μg/ml Ag, (IV) COL–CS–OKGM–Ag hydrogel containing 200 μg/ml Ag + NIR. (**g**) Statistical analyses for the bacterial numbers of *E. coli* and *S. aureus* (mean ± SD, *n* = 3, ***P < 0.001).

The photothermal performance of as-constructed composite hydrogel of COL–CS–OKGM–Ag was studied by irradiation under 808 nm NIR laser with a power of 2 W/cm^2^. After 10 min of irradiation (Fig. 3a), the temperature of the hydrogels containing 200 μg/ml GA–Ag NPs rose rapidly, while the temperature of the hydrogels without silver nanoparticles almost occurred no any changes. For the concentration of 200 μg/ml GA–Ag NPs in the hydrogels, the temperature of the hydrogel increased 21.5°C within 10 min, while the temperature increase of only 7°C for the hydrogels containing 50 μg/ml GA–Ag NPs (Fig. 3b). This difference indicated that GA–Ag NPs have a significant photothermal efficacy, and the hydrogel temperature can be precisely adjusted by controlling the concentration of GA–Ag NPs in these hydrogels. After multiple cycles of irradiation, the maximum temperature of the hydrogel containing 200 μg/ml GA–Ag NPs had no decrease, indicating its good photothermal stability (Fig. 3c). The photothermal conversion efficiency (η) of COL–CS–OKGM–Ag composite hydrogel calculated to be 27.5% (Supplementary Fig. S5), which was higher than some other photothermal agents, such as PVP-Pt NPs (22.99%) [45], Cu_9_S_5_ nanocrystals (25.7%) [46] and Prussian blue nanocages (26%) [47]. The increased temperature of the hydrogel facilitated the release of more Ag^+^ from Ag NPs, which significantly improved the antibacterial capability of the composite hydrogel of COL–CS–OKGM–Ag [48].

### Antibacterial properties of COL–CS–OKGM–Ag hydrogels

*Escherichia coli* and *Staphylococcus aureus* were chosen to evaluate the antibacterial activity of the hydrogels containing Ag NPs, of which the superior photothermal performance enhanced the inherent bactericidal properties of Ag ions alone. This synergistic action not only can effectively prevent bacterial infections but also promote angiogenesis to accelerate wound healing. The group of the COL–CS–OKGM hydrogel did not generate inhibition zones, while the group of the COL–CS–OKGM–Ag hydrogel and the group of the COL–CS–OKGM–Ag hydrogel + NIR produced obvious inhibition areas (Fig. 3d and e). For *E. coli* and *S. aureus*, the diameters of the inhibition zone of the COL–CS–OKGM–Ag hydrogel without NIR irradiation were 2.5 ± 0.52 and 2.4 ± 0.1 cm, respectively. And the diameters of the inhibition zone of the COL–CS–OKGM–Ag hydrogel + NIR were 2.7 ± 0.32 and 2.4 ± 0.1 cm, independently, indicating that the Ag NPs-containing hydrogels had a relatively long-term of at least 12 h antibacterial effect. The Ag^+^ released from the Ag NPs, which can rapidly bind to the negatively charged cell membranes of bacteria, disrupting the normal metabolism of bacteria to result in bacterial death. Compared with the experiment group without NIR, the influence of the photothermal treatments from the group applying NIR on the diameters of inhibition zones was limited over time. From a relatively long-term, the diameter of the inhibition zone mainly depended on the silver ion concentration endowed by the corresponding composite hydrogel containing Ag NPs.

However, from the short-term effects, the auxiliary antibacterial effect of NIR irradiation on the bacterial suspension was prominent in a short time. When *E. coli* and *S. aureus* were in contact with hydrogels for 1 h, the CFU numbers of the bacteria treated with PBS and silver-free COL–CS–OKGM hydrogel, respectively, were almost similar (Fig. 3f). Some specific antibacterial properties were not observed. The hydrogel containing GA–Ag NPs without photothermal treatment exhibited limited antibacterial effect. Although it showed very obvious antibacterial activity during long-term contact, the slow release of Ag^+^ under short-term contact was quite not enough for rapid and effective sterilization. In addition, the CFU number decreased significantly for the group of the hydrogel containing GA–Ag NPs plus NIR (Fig. 3g). This outstanding short-term antibacterial effect was mainly ascribed to the synergistic action of the photothermal efficacy of the GA–Ag NPs and the inherent antibacterial properties of the released Ag^+^, which made up for the insufficient antibacterial effect of Ag^+^ itself within a short period of time and meanwhile maintained the antibacterial activity for a long time.

**Figure 4.**
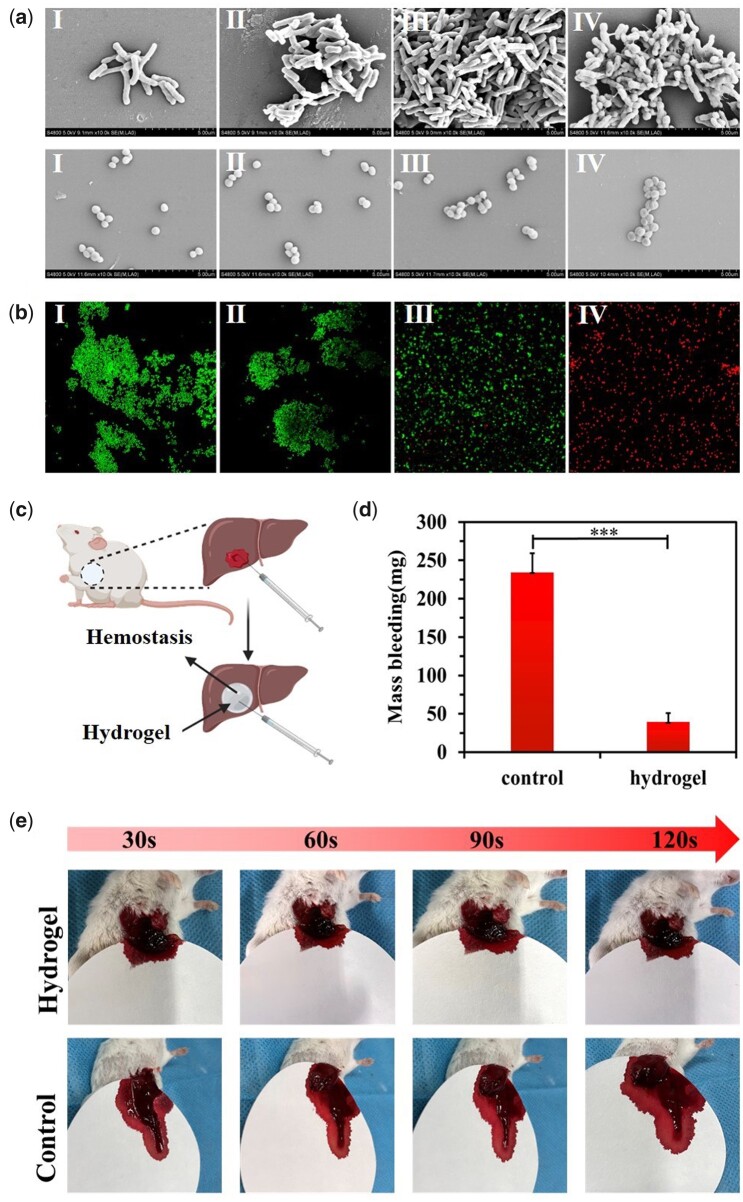
(**a**) SEM images of *E. coli* and *S. aureus* and (**b**) CLSM images of *S. aureus* treated by (I) PBS, (II) COL–CS–OKGM hydrogel, (III) COL–CS–OKGM–Ag hydrogel containing 200 μg/ml Ag NPs, (IV) COL–CS–OKGM–Ag hydrogel containing 200 μg/ml Ag NPs + NIR. PI labeling dead bacteria, and SYTO-9 labeling live bacteria. (**c**) A schematic illustration for the mouse liver hemorrhage model treated by COL–CS–OKGM–Ag hydrogel. (**d**) Accumulated blood loss in 120 s for hepatic hemorrhage under different treatments (mean ± SD, *n* = 3, ***P < 0.001). (**e**) The hemostasis effect of COL–CS–OKGM–Ag hydrogel on damaged mouse liver within 120 s as positive control and untreated group as negative control.

The morphology of the bacteria under different treatments was revealed by SEM to further demonstrate the influence of the GA–Ag NPs within the hydrogel on bacteria (Fig. 4a). The untreated *E. coli* had smooth surface and complete morphology. On the other hand, *E. coli* treated with GA–Ag NPs-contained hydrogel without NIR was slightly damaged. Most of the cell membrane of *E. coli* in the group of COL–CS–OKGM–Ag hydrogel + NIR showed shrinkage and damage. The same changes occurred to *S. aureus*, after irradiated under NIR light, whose cell membrane treated with COL–CS–OKGM–Ag hydrogel showed pits and shrinkage. It was obvious that the COL–CS–OKGM–Ag hydrogel along with NIR irradiation assistance had much better antibacterial activity in a short period of time at initial treatment. The optimum temperature for enzyme activity in bacteria is 30–40°C, and the enzyme activity could be strongly inhibited at higher temperatures. The irradiation of NIR laser promoted the elevation of local temperature and led to bacterial cell membrane destruction and protein denaturation [49]. Simultaneously, the release of Ag^+^ was also accelerated by high temperature and combined with the sulfhydryl groups on the surface of bacteria [50], which can destroy the bacterial cell membrane and thus interact with the nucleic acid to result in bacteria death [51].

As shown in Fig. 4b, there were almost no PI fluorescence in the groups of the bacteria treated with PBS or with the non-silver-containing COL–CS–OKGM hydrogel. A few PI fluorescence spots appeared in the group of the hydrogel containing GA–Ag NPs, indicating that GA–Ag NPs exhibited natural antibacterial ability. When the NIR laser irradiation was performed for 10 min, the bacteria emitted massive PI fluorescence, indicating that most of bacteria had died. The confocal laser scanning microscope (CLSM) images further confirmed that the synergy between the photothermal properties of GA–Ag NPs and the inherent bactericidal activity of Ag^+^ during the short-term contact can destroy the integrity of cell membranes and induce the death of bacteria.

### Evaluation of *in vivo* hemostatic effects of COL–CS–OKGM–Ag hydrogel

Infection from wound bleeding is one of the main causes of tissue complications, leading to inflammation and delayed wound healing [52]. Timely hemostasis is the first step in wound repair. Therefore, mouse liver hemorrhage models were applied to evaluate the hemostatic ability of the COL–CS–OKGM–Ag hydrogel (Fig. 4c). First of all, from Fig. 4e, it was obvious that due to a puncture on the liver, the blood seeped out from the pinhole, which will flow out continuously if no effective treatment was adopted. In contrast, when the hydrogel was injected onto the wound area immediately after the occurrence of bleeding, the bleeding was controlled and stopped within 30 s. As shown in Fig. 4d, 2 min after the hydrogel injection onto the liver wound, the bleeding volume was 39.33 ± 11.4 mg, while the bleeding volume from the untreated liver wound reached up to 234.33 ± 24.7 mg. The rapid hemostatic ability of the COL–CS–OKGM–Ag hydrogel should be attributed to the excellent performance of collagen and chitosan. Positively charged collagen could attract platelet aggregation and activate intrinsic pathways of the secondary hemostatic process. Chitosan could agglutinate with negatively charged erythrocytes and effectively activate platelets to achieve good hemostasis efficacy [53].

### *In vitro* biocompatibility evaluation for hydrogel of COL–CS–OKGM–Ag

**Figure 5.**
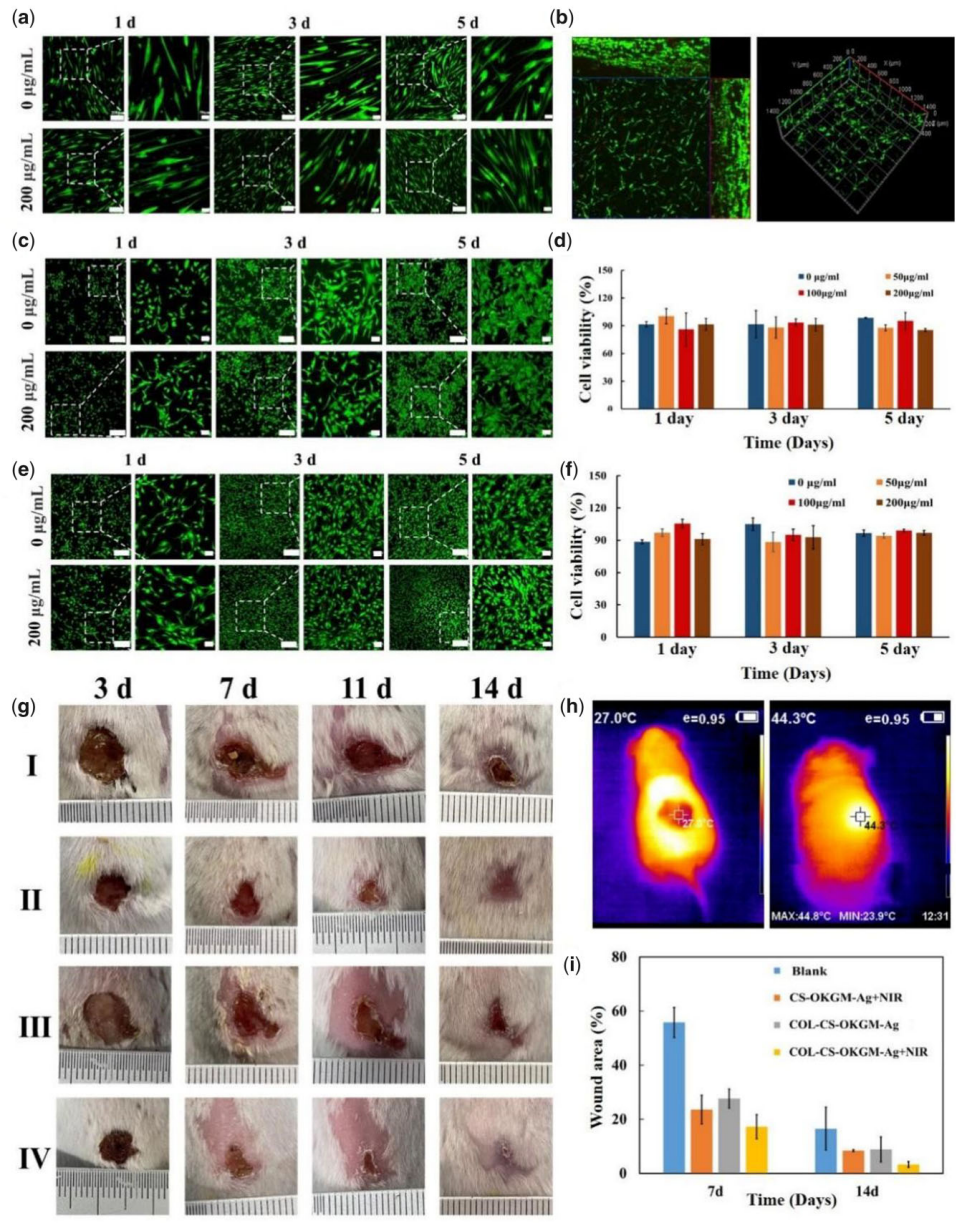
CLSM images of (**a**) HDF cells, (**c**) HUVEC and (**e**) NIH3T3 cells cultured on the surface of COL–CS–OKGM–Ag hydrogel containing from 0 to 200 μg/ml Ag NPs (bar = 200 μm, 50 μm). (**b**) Three-dimensional reconstruction of CLSM image of HDF cells embedded and cultured inside the hydrogel for 5 days. Cells were cultured on hydrogels with different silver content for 1, 3 and 5 days, the proliferation of (**d**) HUVECs and (**f**) NIH3T3 cells was detected by CCK-8. (**g**) Photographs of *S. aureus* infected wounds at predetermined time points for different treatment groups. (**h**) Temperature changes showed by thermal infrared images of mice irradiated by infrared laser and (**i**) percentage of wound area *in vivo* at 7 and 14 days.

Firstly, human dermal fibroblasts (HDF) cells were cultured on the surface of COL–CS–OKGM–Ag hydrogels (Fig. 5a). After 24 h, whether for the silver-free hydrogels or the COL–CS–OKGM–Ag hydrogels containing 200 μg/ml Ag NPs, these spindle-shaped cells had adhered to the surface of the hydrogels and diffused well. An important sign of early cell proliferation is the extension of membranous processes such as filopodia or lamellar feet [54]. HDF cells produced visible filopodia and formed slender spindle shape to effectively promote cell proliferation. After 3 or 5 days of cultivation, the cells showed evident proliferation. In addition, HUVEC cells and NIH3T3 cells were also inoculated on the surface of hydrogel (Fig. 5c and e), respectively. Both HUVEC and NIH3T3 cells could attach to the hydrogel within 1 day and proliferated over the next 4 days. Compared with the blank group, the cells cultured on the hydrogels with different silver content all showed contiguous cell viability (Fig. 5d and f). The cell viability of the COL–CS–OKGM–Ag hydrogel groups remained above 85% after incubation for 1, 3 and 5 days. To further verify the cell compatibility of so-designed hydrogel material, HDF cells (Fig. 5b) and NIH3T3 cells (Supplementary Fig. S6) were co-mixed with the hydrogel precursor solution before gelation, respectively, thus to culture these cells inside the hydrogel. After 5 days of culture, HDF and NIH3T3 cells proliferated and grew significantly inside the hydrogel with few dead cells. Then histological analyses were performed on the major organs of mice treated with the hydrogel material for 14 days (Supplementary Fig. S8). All tissues showed a normal structural state without obvious organ damage and inflammation. These results confirmed the excellent biocompatibility of COL–CS–OKGM–Ag hydrogels, which can provide a suitable microenvironment for cell survival and growth. According to ISO/TR 7406 [55], hemolysis ratio (<5%) for the COL–CS–OKGM hydrogels with different silver concentration is in the critical safe range of hemolysis for biomaterials, suggesting the good blood compatibility (Supplementary Fig. S7).

### *In vivo* evaluation for wound healing

According to wet-wound healing theory, COL–CS–OKGM–Ag hydrogel, as a water-rich dressing containing bioactive macromolecules and Ag nanoparticles, assisted complex wounds including infected wounds with regenerative healing. A full-thickness wound of a round incision with a diameter of 1.0 cm on the back of the mouse, which was infected by *S. aureus*, was constructed as an infection wound model. The degree of wound healing in each group of mice (Fig. 5g) was recorded by camera at different times. Under the irradiation of 808 nm NIR light for 1 min (Fig. 5h), the local temperature of the wound area, which was covered by the COL–CS–OKGM–Ag hydrogel, rose rapidly from 27°C to 44°C. The wound area was maintained at a mild temperature of <45°C without burning the skin by controlling the power of the NIR light emitter. The results showed that this COL–CS–OKGM–Ag hydrogel could be used as a good PTT for human wound healing. After 7 days of treatment, compared with the untreated group, the wound area of the treated group decreased to a higher degree. After 14 days of treatment, the new epidermis gradually extended to the center and covered the former wound area. During the recovering period, the group of COL–CS–OKGM–Ag + NIR hydrogel exhibited the fastest healing process compared with the other groups, of which new growth skin was very similar to the normal skin. The wound area decreased only to 3.2% on the 14th day (Fig. 5i), while the wounds of the blank group still had obvious scabs. Such designed hydrogel dressings based on collagen and chitosan had been proved to have expected potential of promoting wound healing [56]. This COL–CS–OKGM–Ag hydrogel combining NIR laser irradiation had effectively increased antibacterial activity on infectious wounds with accelerating the wound healing process due to the thermal therapy efficacy from Ag nanoparticles.

**Figure 6.**
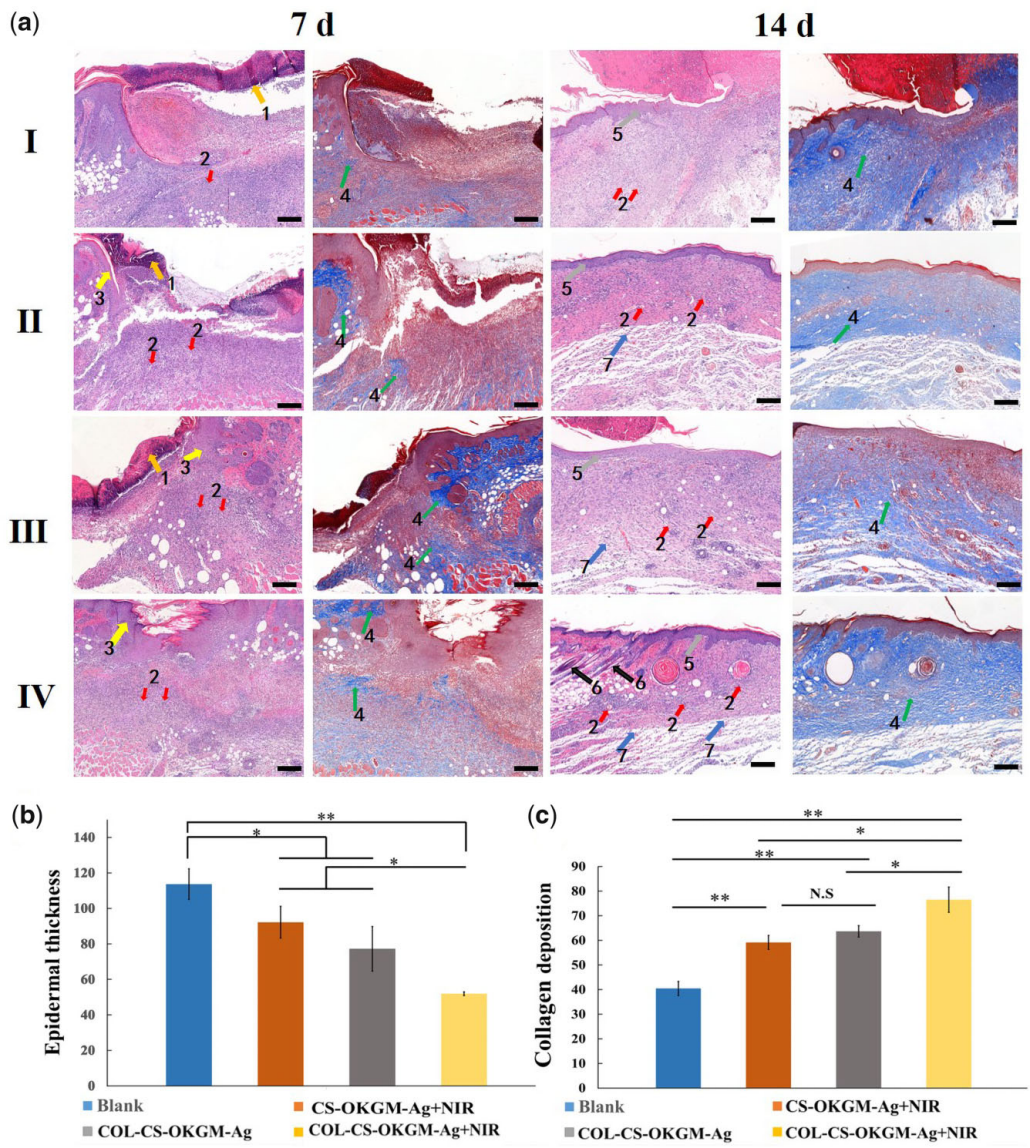
(**a**) H&E staining and Masson staining of the wound sites on 7th and 14th day. Scale bars = 200 μm. Arrow 1: scab skin; Arrow 2: blood vessels; Arrow 3: thickened epidermis; Arrow 4: collagen fiber; Arrow 5: epidermal tissue; Arrow 6: hair follicle; Arrow 7: fibroblasts (*n* = 3). (**b**) The epidermal thickness and (**c**) collagen deposition was measured on 14th day (mean ± SD, *n* = 3, **P < 0.01).

Histological analyses further evaluated the regenerative skin tissue of the hydrogel-treated infected wound. Inflammatory cell infiltration still existed in the wound area on the seventh day (Fig. 6a), while the wound edge was in healing and the new epidermis became thicker compared with the surrounding normal skin. Fine neovascularization appeared in the lower layer of the wound. On the 14th day, it can be seen that the wound of the hydrogel treatment group has healed basically and formed a complete epidermis layer. Both the epithelium and connective tissue were shown to have greater regularity, and new hair follicles were obvious at the wound site, which implied the functional regenerative repair of the skin tissue. The epidermal thickness of the wound treated with COL–CS–OKGM–Ag + NIR was thinner than that of the other three groups (Fig. 6b). Since epidermal thickening is related to hypertrophic scar formation [57], it was further suggested that COL–CS–OKGM–Ag hydrogel plus photothermal treatment reduced the formation of scars. In addition, in the process of wound repair, collagen is a necessary condition for wound healing and dermal reconstruction [58]. In Masson’s three-color staining slices, a small amount of collagen deposition occurred in the treatment group on Day 7 and the area of new collagen fibers increased greatly on the 14th day. Compared with the other groups, the wounds treated by the group of COL–CS–OKGM–Ag + NIR hydrogel showed more collagen deposition (Fig. 6c), which were dense and orderly. These results had demonstrated that the group of COL–CS–OKGM–Ag + NIR exhibited better healing effect than other groups, because collagen deposition and granulation formation were positively correlated with the state of wound healing [59].

**Figure 7.**
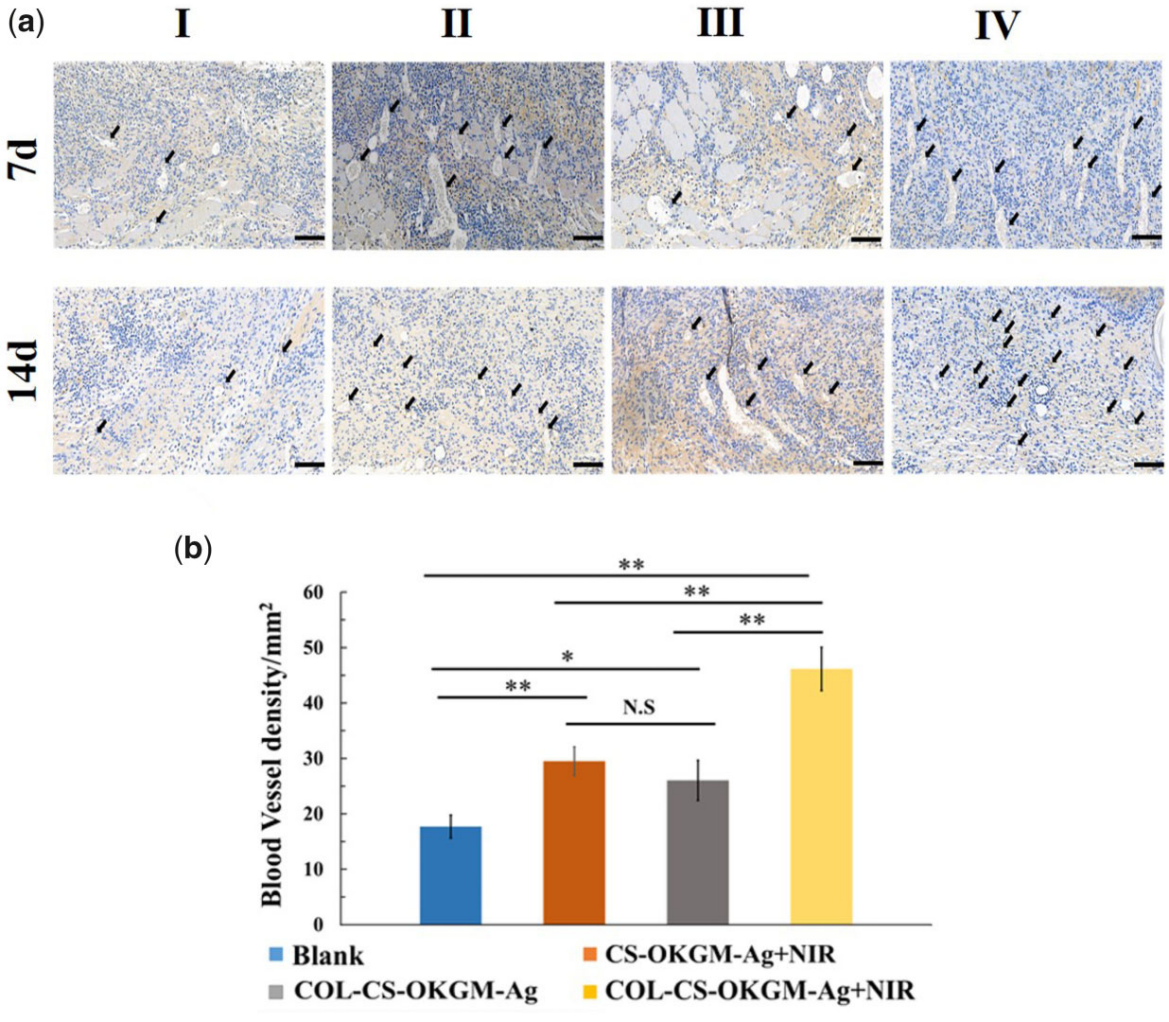
(**a**) CD31 immunohistochemically staining on the 7th and 14th day of the wound treatment. Scale bars =100μm, black arrows: blood vessels. (**b**) Number of newly formed blood vessels from the immunohistochemical images on Day 14 (mean ± SD, *n* = 3, **P < 0.01). Note: I: blank; II: CS–OKGM–Ag hydrogel + NIR; III: COL–CS–OKGM–Ag hydrogel; IV: COL–CS–OKGM–Ag hydrogel + NIR.

Wound healing needs new blood vessels to transport nutrients and metabolism to achieve reconstruction for tissue repair [60]. Therefore, angiogenesis is also an important indicator for wound healing evaluation [61]. As showed by CD31 immunohistochemical analyses (Fig. 7a), on the seventh day, new vessels already appeared in the wound area for different groups, with fewest new vessels in the blank group while most vessels in the COL–CS–OKGM–Ag + NIR treated group. On Day 14, there were more new vessels in the VI group than in the control group (Fig. 7b). These phenomena indicated that the COL–CS–OKGM–Ag hydrogel plus mild thermal stimulation can facilitate the neovascularization. Remarkably, the healing process of infected wounds and the regeneration of new blood vessels are slow and persistent, so the irradiation of NIR light in animal experiments is also a long-term regular process to ensure effective killing of bacteria and stimulation of angiogenesis. The syringeability and the self-healing properties of the COL–CS–OKGM hydrogels enable this dressing type to fully fit to irregular wound surface, providing an appropriately moist environment for healing process. The antibacterial effect of the COL–CS–OKGM–Ag hydrogel can be synergistically enhanced by the photothermal function of the Ag NPs, which meanwhile can give mild photothermal stimulation for accelerating wound healing in turn.

## Conclusions

A multifunctional hydrogel dressing integrating antibacterial and hemostasis activities together with mild PTT was smartly designed for treating complicated wounds by injecting this hydrogel on wound area to maintain a moist environment for regenerative healing rather than cicatrix repair. This collagen-based hydrogel was successfully constructed through incorporating Ag-NPs into COL–CS–OKGM hydrogel matrix, which was confirmed by rheological characterization for its excellent injectable and self-healing behaviors originating from reversible Schiff-base linkages. *In vitro* and *in vivo* evaluations demonstrated the good biocompatibility of the COL–CS–OKGM–Ag hydrogel and the markedly enhanced antibacterial action provided by the synergistical effect of Ag^+^ and mild photothermal efficacy of Ag NPs, which improved the local capillary circulation of the wound area to further accelerate wound healing process. Meanwhile, this hydrogel matrix of COL–CS–OKGM–Ag has also been demonstrated to be an excellent hemostasis material for inhibiting wound bleeding. The injectable self-healing properties make the COL–CS–OKGM–Ag hydrogel a convenient dressing material for the wounds with irregular and large area needing frequent applying and changing without secondary injury. So-designed composite hydrogel is a promising multifunction platform for wound therapies with great safety and desired regenerative prognoses.

This study emphasizes the proof-of-concept applications of such multifunctional hydrogel for the treatment of various wounds derived from some diseases or tumor resection. But as many reported relevant studies, our works were conducted only on infectious wound models. Further investigations based on more complicated wound models will be necessary to confirm the adjuvant therapy and management strategy for the regenerative healing achieved by this multifunctional hydrogel dressing.

## Supplementary Material

rbad018_Supplementary_Data
