# Supplementary material for: Collagen-based injectable and self-healing hydrogel with multifunction for regenerative repairment of infected wounds
Source: Regen Biomater. 2023 Mar 7;10:rbad018. doi: 10.1093/rb/rbad018 (PMC10039733; doi:10.1093/rb/rbad018)
Supplement: rbad018_Supplementary_Data [file rbad018_supplementary_data.docx]

**Supplementary Information**

**Collagen-based injectable and self-healing hydrogel with multifunction for regenerative repairment of infected wounds**

Haojie Gu^1,2^, Han Li^1,2^, Liren Wei^3^, Jian Lu^1,2^ and Qingrong Wei^1,2,^*

^1^ *National Engineering Research Center for Biomaterials (NERCB), Sichuan University, Chengdu 610065, P. R. China*

^2^ *College of Biomedical Engineering, Sichuan University, Chengdu 610065, P. R. China*

^3^ *School of Life Science and Engineering, Southwest University of Science and Technology, Mianyang 621000, P. R. China*

* Corresponding author. *E-mail address:* qingrongwei@scu.edu.cn (Q. Wei)

1. **Experimental Details**

***1.1*** ***Swelling test of hydrogel***

The swelling performance of hydrogel was evaluated by calculating the weight change. First, each lyophilized hydrogel (W_0_) of known weight was immersed in PBS (pH=7.4) at room temperature. At different time points, the hydrogel was taken out and gently absorbed the excess water on the surface with filter paper. Then weigh the weight of the hydrogel (W_s_). The swelling ratio is calculated according to the following formula:

Swelling ratio(%)=(Ws-W_0_)/W_0_ *100

***1.2*** ***Dehydration test of hydrogel***

The dehydration test of hydrogel was carried out. Fresh hydrogels of uniform thickness were prepared in 24 well plates and weighed (W_W_). The hydrogel was placed in a constant temperature and humidity air shaker (37℃, humidity :50wt%) and its weight (W_t_) was recorded every 1.5 h. When the weight was constant, it was set as dry weight (W_d_). The moisture content is calculated as follows:

water content(%)=( W_t_ - W_d_)/( W_W_- W_d_) *100

***1.3 Hemolysis test of hydrogel***

Red blood cells (RBCs) were collected from mouse blood (female Kunming mice, 25-30 g) by centrifugation (1500 rpm 10 min). Then, RBCs was diluted with PBS (pH 7.4). RBCs dispersions treated with H_2_O were used as the positive control (PC), and RBCS dispersions treated with PBS were used as the negative control (NC). The hydrogel sample (0.1 g) was then dipped into the RBCS dispersion (1.5 mL). After 3 h, the absorbance of the supernatant at 541 nm was measured by ultraviolet spectrophotometer. Hemolysis rate (%) was calculated as follows:

Hemolysis rate (%) =(A _sample_ -A _NC_)/(A _PC_ -A _NC_) * 100.

***1.4 Measurements of*** ***photothermal performance in vitro***

To study the photothermal effects of as-constructed hydrogel of COL-CS-OKGM-Ag, 1.5 mL of this hydrogel was injected into a vial and exposed to 808 nm NIR laser at a power density of 2 W/cm^2^ for 10 min. Hydrogels containing 0 μg/mL, 50 μg/mL, 100 μg/mL, 150 μg/mL, 200 μg/mL GA-Ag NPs were placed under 808nm NIR laser (2 W/cm^2^ ) irradiation, respectively. The hydrogel containing 200 μg/mL GA-Ag NPs was irradiated with three cycles comprising of NIR laser-irradiation heating (808 nm, 2 W/cm^2^, 10 min) and natural cooling to evaluate its photothermal stability. During these processes, the temperature variations were recorded with a thermal imaging camera (FLIR T460) at an interval of 0.5 min.

***1.5 Rheological characterization***

The rheologic behaviour of as-prepared composite hydrogel of COL-CS-OKGM-Ag was necessary to be investigated to identify its smooth injectability through syringe needle, as well as including its self-healing ability. The rheological measurements were performed at 37℃ on a modular compact rheometer (MCR102, Anton Paar, Germany) using the storage modulus (G′) and loss modulus (G″) under different oscillation, strain and shear rate. In the strain range of 0.1-1000 %, the breaking strain of the hydrogel was evaluated under a frequency of 1 Hz. In the step-strain tests, the stains of 1 % and 500 % were alternately applied to the hydrogel with 3 cycles for 120 s at a constant frequency of 1.0 rad/s. In the shear rate range of 0.1-300 1/s., the impact of high shear rate on the viscosity of the hydrogel was measured.

***1.6*** ***Antibacterial activity in vitro***

Using the spread plate method, the antibacterial properties of the hydrogel were analyzed with *S. aureus* and *E. coli*. First, 100 µl of bacterial solution (10^8^ CFU /mL) was added dropwise to 100 µl of PBS solution as a blank sample. At the same time, the bacterial liquid was dropped on the surface of COL-CS-OKGM hydrogel with or without silver nanoparticles as a control group. The experimental group was designed as the hydrogel of COL-CS-OKGM-Ag plus the irradiation under 808 nm laser (2 W/cm^2^, 10 minutes) , which is labelled as COL-CS-OKGM-Ag + NIR for all the following experiments. After incubating the hydrogels of all groups in the bacterial suspension for one hour, the hydrogel material was washed with 900 µl of PBS, and 100 µl of the suspension was spread on the [agar plate](https://www.sciencedirect.com/topics/engineering/agar-plate) (Petri dish). After incubation for 24 hours, the CFU number of bacteria was counted on the agar plate. [In](javascript:;) [addition](javascript:;), the experiment of bacteriostatic zone was carried out to further evaluate the continuity of the hydrogel’s bacteriostasis. 200 µl of *S. aureus* and *E. coli* (10^6^ CFU /mL) were evenly spread on the LB agar plate individually, then the silver-free and silver-containing hydrogels were placed on the agar plate, respectively. One of the COL-CS-OKGM-Ag hydrogel was irradiated by 808 nm laser (2 W/cm^2^, 10 minutes), while the other one was left untreated. After incubation for 24 hours in bacterial incubator , the diameter of the inhibition zone was measured.

***1.7 Bacterial Morphological Analysis***

Fluorescent live-death staining was used to verify the integrity of the bacterial cell membrane. For the experimental group, 100 µl of bacterial suspension (10^8^ CFU /mL) was dropped on the surface of COL-CS-OKGM-Ag hydrogel, which then irradiated with 808 nm laser (2 W/cm^2^) for ten minutes. And for the other groups, 100 µl of bacterial suspension was dropped in PBS and on the COL-CS-OKGM and COL-CS-OKGM-Ag hydrogel, respectively. After one hour of incubation, the bacteria on the hydrogels were rinsed with 900 µl PBS, from which 100 μl solution was taken to mix with 100 µl of bacterial viability dye (Thermo Scientific Molecular Probes' LIVE/DEAD® BacLight TM Bacterial Viability Kits，SYTO 9, 0.01 mM, 50 µl; propidium iodide (PI), 0.06 mM, 50 µl), incubating in dark for 15 minutes. All samples were observed under laser scanning confocal microscope (CLSM, Zeiss Ism710, Germany). Live cells were green by SYTO 9 staining, and dead cells were red by PI staining. Subsequently, the morphological changes of the bacteria after different treatments were observed under scanning electron microscope (SEM). The bacteria were collected from the bacterial suspension after the treatment with PBS, which was fixed with 2.5% glutaraldehyde solution overnight and then dehydrated with gradient ethanol solution (30, 50, 70, 90 and 100%, v/v) for 10 minutes [individual](javascript:;)ly. After dried in carbon dioxide critical point drier, all samples were imaged by the field-emission scanning electron microscope (FE-SEM, S-4800, Hitachi, Japan) with an accelerating voltage of 10 kV.

***1.8 Cytotoxicity evaluation***

Mouse embryonic fibroblast (NIH-3T3), [Human](javascript:;) [umbilical](javascript:;) [vein](javascript:;) [endothelial](javascript:;) [cell](javascript:;) (HUVEC) and Human dermal fibroblasts(HDF) were utilized to evaluate the cytotoxicity of the hydrogel. The hydrogels were spread with different silver concentrations (0 µg/mL、50 µg/mL、100 µg/mL、150 µg/mL、200 µg/mL) on the bottom of the 24-well plate, and then NIH-3T3 cells, HUVEC cells and HDF(1×10^5^) were seeded [respective](javascript:;)ly on the surface of the hydrogels. Afterward, these hydrogels with cells were incubated in culture for 1, 3 and 5 days. The NIH-3T3, HUVEC and HDF cells images of 200 µg/mL Ag-containing hydrogel were recorded by CLSM and CCK-8 was used for quantitative analyses. Take the cells undergoing no any treatments as the positive control (PC), and the blank wells without any cells as the negative control (NC). The relative cell viability is calculated as follows:

cell viability= (OD sample-OD NC) / (OD PC-OD NC) *100%

To further identify the biocompatibility of the composite hydrogels, NIH-3T3 and HDF cells were mixed with the liquid precursor to be embedded into the hydrogel, respectively, then was cultured in DMEM medium for 5 days. FDA and PI were used as live and dead staining, and the images for three-dimensional culturing were obtained by CLSM .

***1.9 In vivo hemostatic performance of hydrogels***

The hemostatic ability of hydrogel *in vivo* was studied by mouse hemorrhagic liver model (female Kunming mice, 25-30 g). The abdominal cavity was cut to expose a part of the liver, which was induced bleeding by 18 G needle. A piece of pre-weighed filter paper was put under the liver and then hydrogel was injected into the injured area. The photos were recorded every 30 seconds and the weight of the filter paper was measured after 120 seconds to calculate the amount of bleeding.

***1.10 In vivo antibacterial and wound healing***

The female Kunming mice (18-22 g) of 6~8 weeks were purchased from Chengdu Dossy Experimental Animal Co, Ltd. The mice were randomly divided into 4 groups, blank group (PBS treatment), control group (200 µg/mL Ag-containing CS-OKGM hydrogel plus near-infrared irradiation), 200 µg/mL Ag-containing COL-CS-OKGM hydrogel group and 200 µg/mL Ag-containing COL-CS-OKGM hydrogel plus near-infrared irradiation group. 200 µl of *S. aureus* liquid (10^8^ CFU /mL) was dropped on a 10mm full-thickness skin lesion to establish a mouse wound infection model. After 24 hours, the hydrogel was injected to cover the wound area, and the illuminated group was irradiated with 808nm NIR laser (10 minutes, 2 W/cm^2^). The hydrogel was replaced every two days for a total of seven times, and photothermal treatment was performed every time when the hydrogel was changed in the illuminated group. After a treatment period of 14 days, the wound healing was screened, and the tissue around the wound was taken for pathological analyses.

**2. Results**

**Table S1** **The molecular weights of KGM and OKGM**

| **Simple** | **Mn** | **Mw** | **PDI** |
| --- | --- | --- | --- |
| **OKGM** | **46037** | **72857** | **1.58** |
| **KGM** | **837999** | **1026941** | **1.22** |


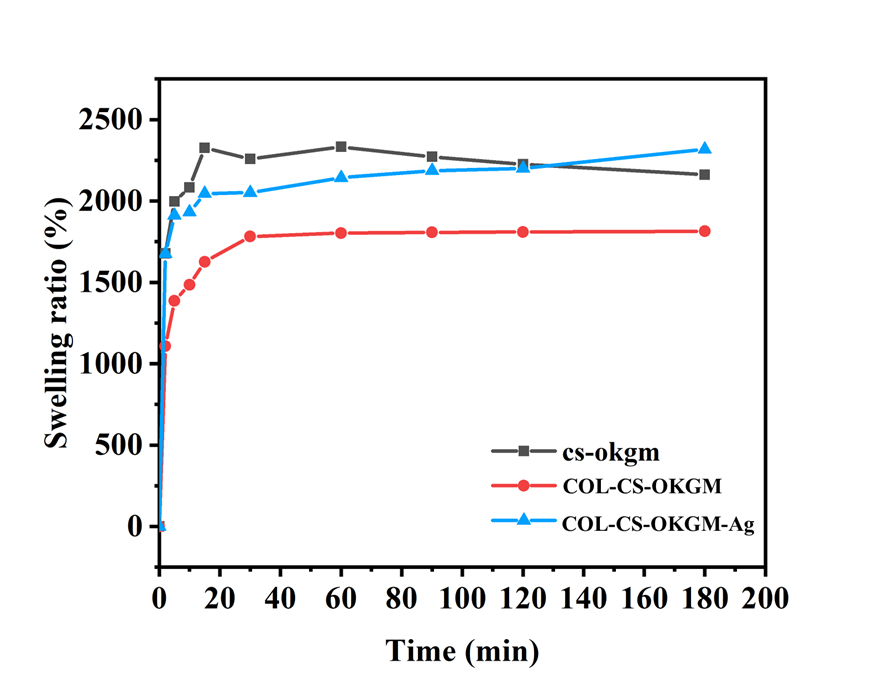


Fig.S1. Swelling rate of CS-OKGM, COL-CS-OKGM, COL-CS-OKGM-Ag hydrogels.


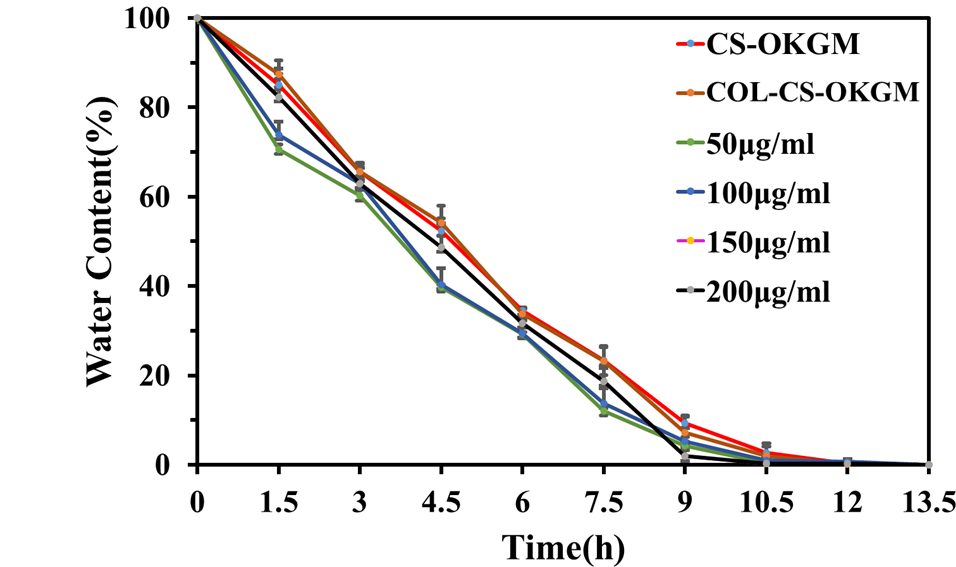


Fig.S2. Water content curves of CS-OKGM hydrogels and COL-CS-OKGM hydrogels with different Ag NPs concentrations.


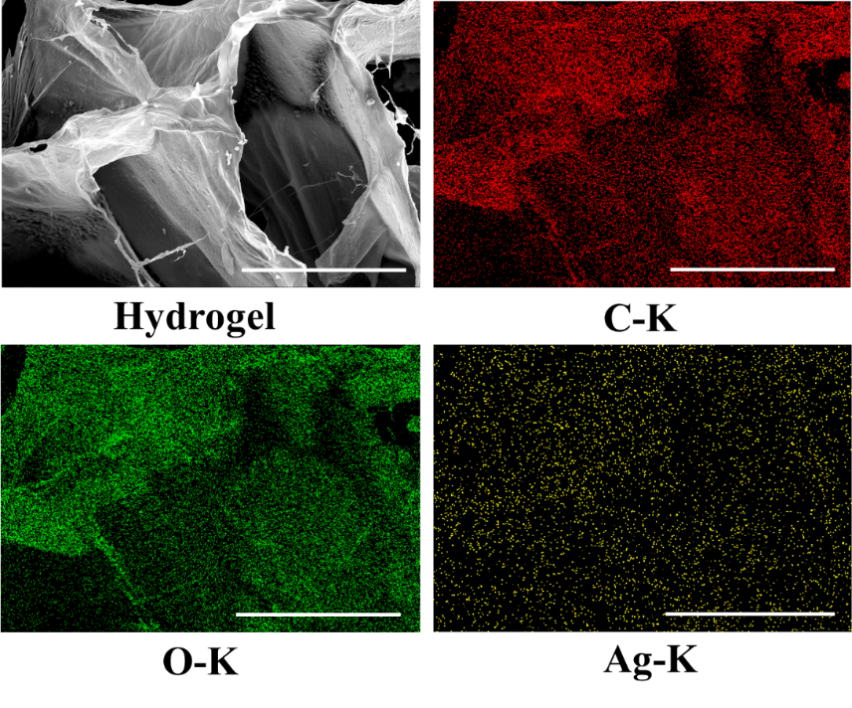


Fig.S3. Mapping of C, O and Ag elements in COL-CS-OKGM-Ag hydrogel (200 μg/ mL).


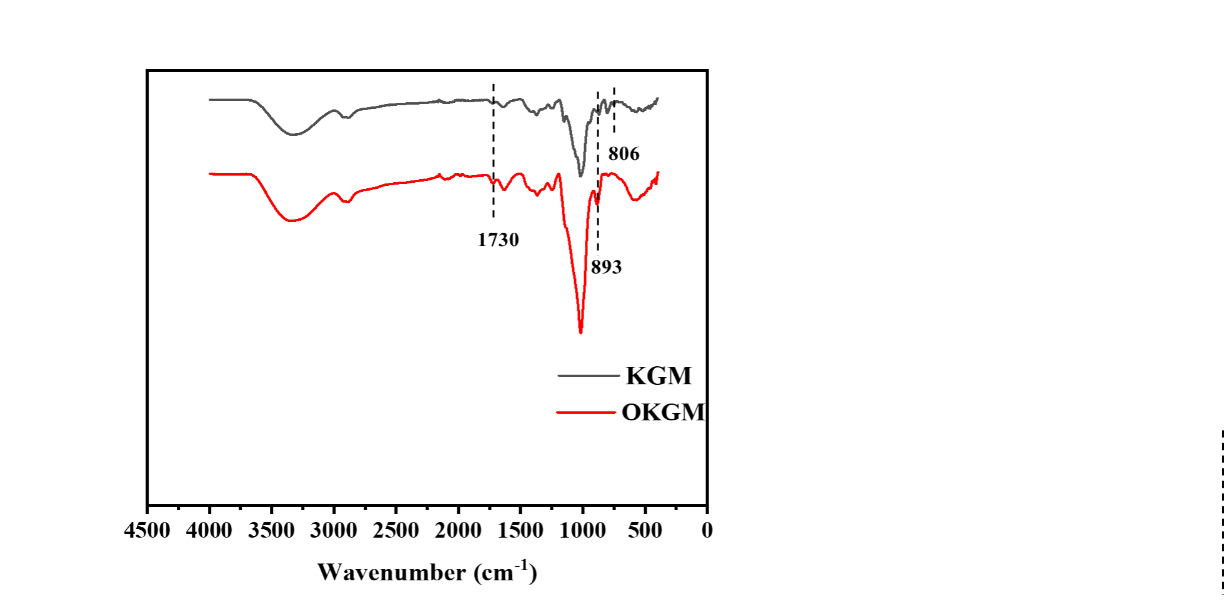


Fig.S4. FTIR spectra of KGM and OKGM


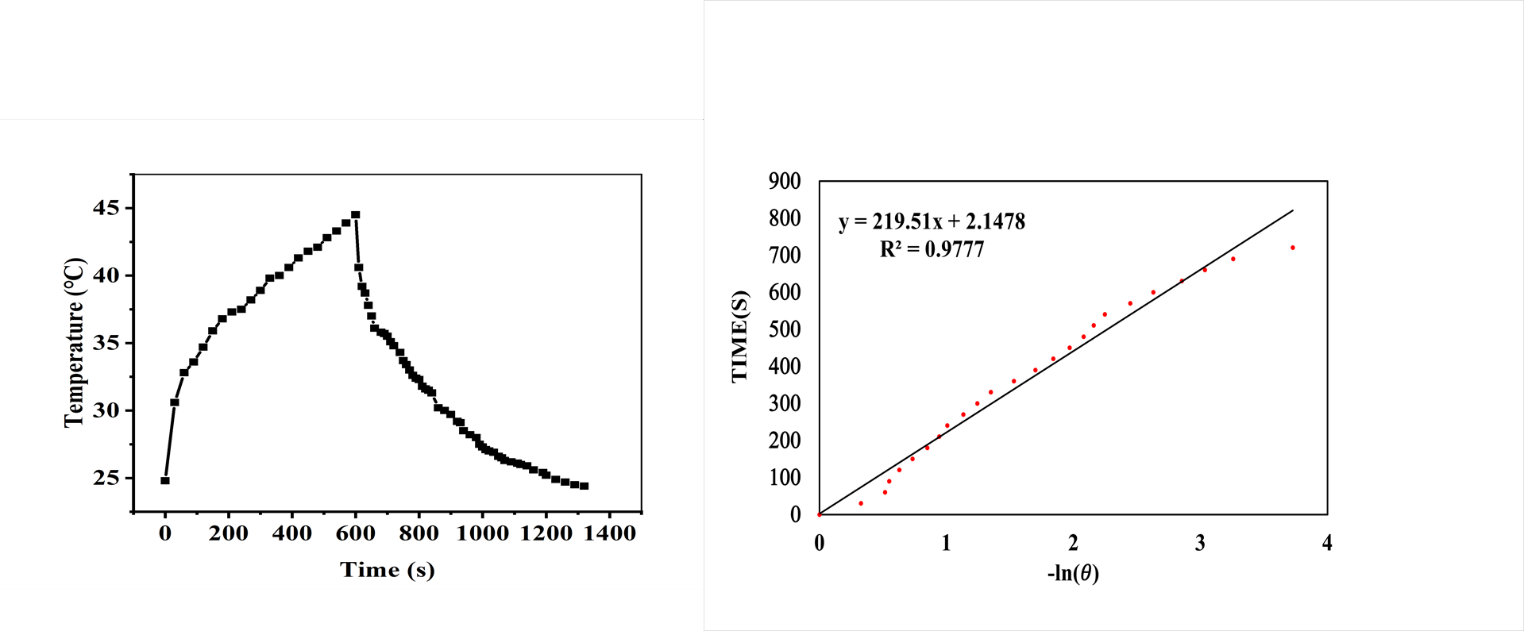


Fig.S5. According to the heating - cooling curve of COL-CS-OKGM-Ag hydrogel in one cycle, the time constant (τ) of heat transfer of hydrogel was determined by linear fitting of the time data of cooling cycle with the negative natural logarithm of temperature.


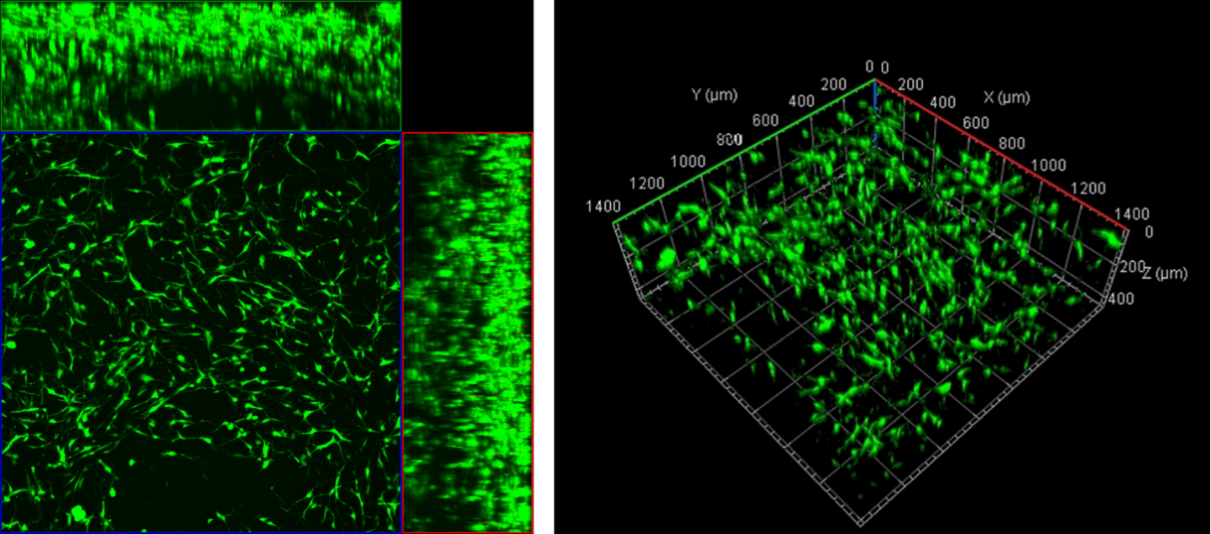


Fig.S6. 3D reconstruction of confocal laser scanning micrograph image of NIH3T3 cells cultured in hydrogel for five days.


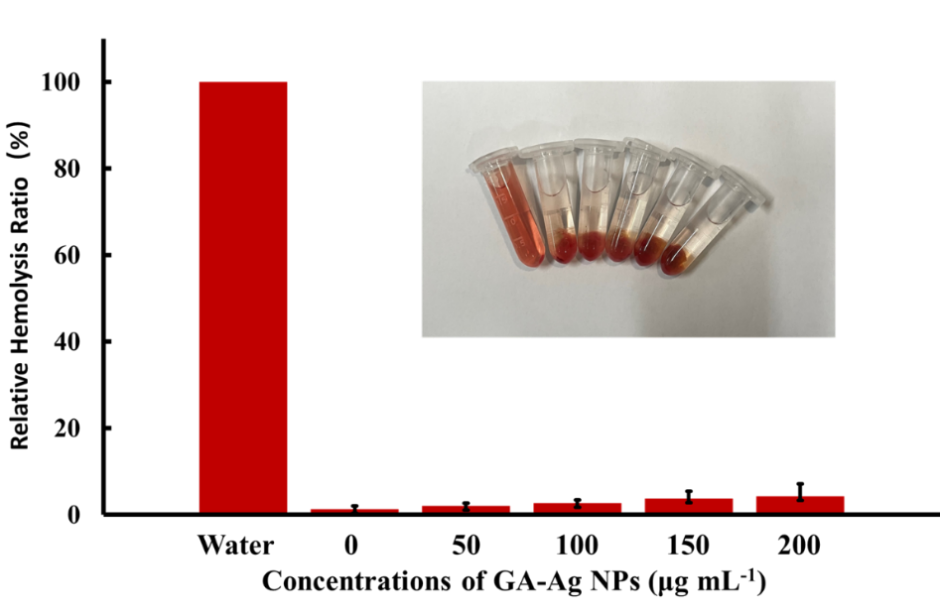


Fig.S7. Relative hemolysis rate of hydrogels containing different concentrations of GA-Ag NPs. The attached figure is the hemolysis test chart.


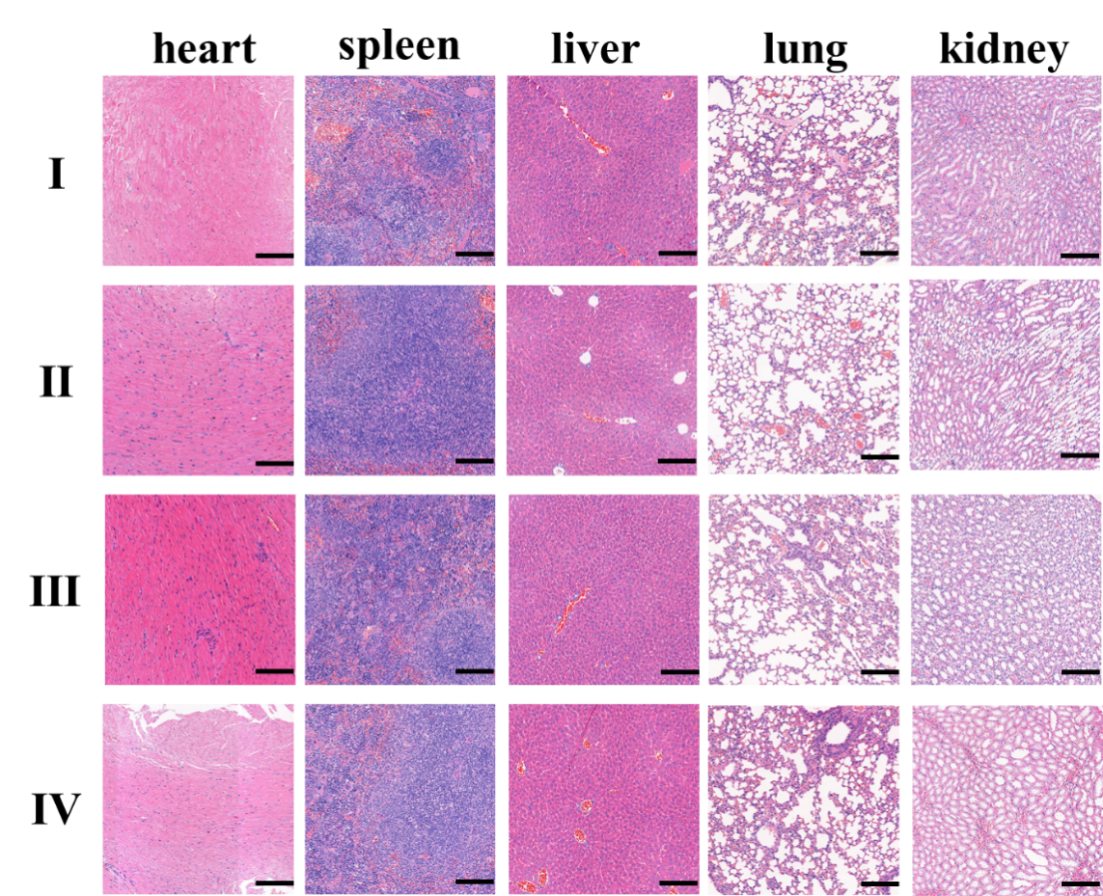


Fig.S8. Biocompatibility test of COL-CS-OKGM-Ag hydrogel. Histological analysis of the major organs (heart, liver, spleen, lungs, and kidneys) was performed 14 days after wound treatment (H&E). The bar = 200µm . Note: Ⅰ:the blank; Ⅱ: CS-OKGM-Ag hydrogel + NIR; Ⅲ: COL-CS-OKGM-Ag hydrogel; Ⅳ: COL-CS-OKGM-Ag hydrogel + NIR.
